# Nitrofurantoin-Induced Exfoliative Dermatitis: A Case Report

**DOI:** 10.7759/cureus.29570

**Published:** 2022-09-25

**Authors:** Carolina Morna, Jéssica Chaves, Carolina Barros, Tiago E Freitas

**Affiliations:** 1 Internal Medicine, Hospital Dr. Nélio Mendonça, Funchal, PRT

**Keywords:** hypersensitivity reaction, drug reaction, nitrofurantoin, exfoliative dermatitis, erythroderma

## Abstract

Exfoliative dermatitis (ED) is a rare and life-threatening dermatological emergency caused by a wide range of cutaneous or systemic conditions, such as inflammatory dermatosis, drug reactions, and malignancies. We report a case of a 77-year-old man who developed ED five days after starting nitrofurantoin. The drug was withdrawn, and the patient was treated with topical corticosteroid and supportive care, after which there was a full recovery within a week. This report describes an uncommon entity with a guarded prognosis that requires proper diagnosis and management.

## Introduction

Exfoliative dermatitis (ED), also known as erythroderma, is a severe and rare disease characterized by diffuse erythema and scaling of more than 90% of the body surface [1-3]. It is more common in elderly males, and the disease may be caused by a variety of underlying causes, such as dermatoses, infections, drugs, and systemic diseases. Drug hypersensitivity reactions are the second most frequent cause of ED [2].

Nitrofurantoin is an antibacterial drug frequently used in the treatment of uncomplicated cystitis that is usually well tolerated. Adverse effects are commonly mild and include gastrointestinal complaints, headache, and dizziness. Isolated case reports have documented severe hypersensitivity reactions [4].

Since ED is a dermatological emergency, it is essential to recognize and manage this condition appropriately [2,3].

## Case presentation

A 77-year-old man, with a previous history of type 2 diabetes mellitus (DM) and suprapubic catheterization due to a severe urethral stricture, presented to the emergency department with multiple erythematous, scaly, desquamative, and itchy skin lesions. The lesions had two days of evolution with a rapid onset, and affected more than 90% of his body surface area, including all four limbs, face, and trunk. The patient was recently diagnosed with a urinary tract infection and started oral nitrofurantoin 100mg every six hours five days before.

**Figure 1:**
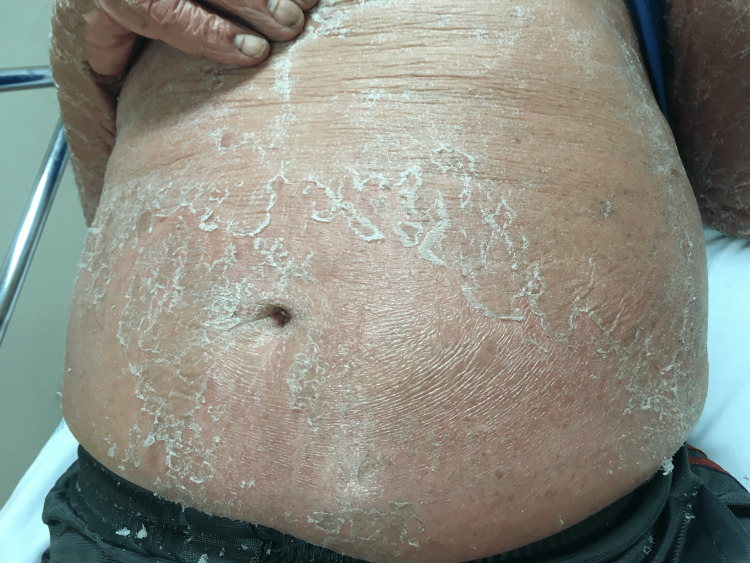
Exfoliative dermatitis with exfoliation and scaling on the abdomen

**Figure 2:**
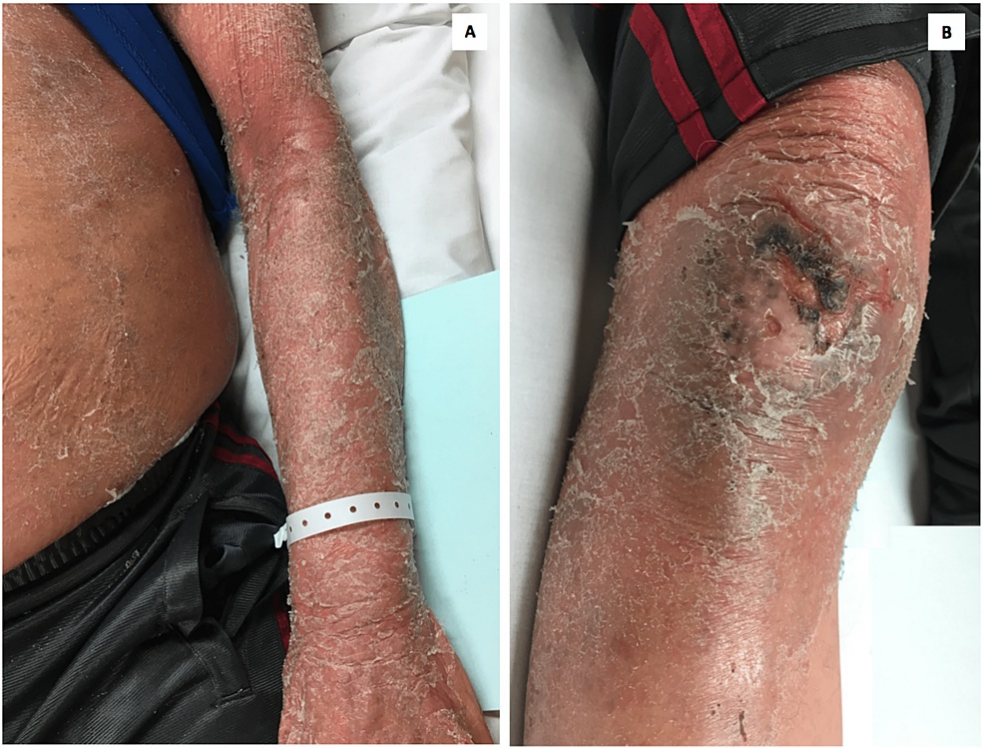
Severe exfoliation and scaling over the upper limbs (A) and lower limbs (B)

On admission, he was eutrophic, with a temperature of 36.8ºC, blood pressure of 123/68 mmHg, and heart rate of 88 bpm. There was neither visceromegaly nor lymph node enlargement. He had erythematous desquamative confluent patches distributed over his face, neck, trunk (Figure 1), and limbs (Figure 2), involving flexural areas but sparing palms, soles, and mucous membranes.

He had chronic mild anemia with hemoglobin of 11.4 g/dL, hematocrit of 41.2%, leukocytosis, with a normal platelet count. Creatinine levels were slightly above the upper limit, and C-reactive protein was increased. Laboratory findings are described in Table 1.

**Table 1:** Laboratory analyses LDH: lactate dehydrogenase

Parameter	On admission	Fifth day	Reference values
Hemoglobin (g/dL)	11.4	10.9	13.7-17.3
Hematocrit (%)	41.2	38.7	40-51
Leucocytes (x10^3^ μL)	16.5	9.7	4.2-10.8
Eosinophils (x10^3^ μL)	0.6	0.4	0.05-0.5
Platelets (x10^3^ μL)	352	301	144-440
Creatinine (mg/dL)	1.32	1.13	0.7-1.2
LDH (U/L)	266	198	0-246
C-reactive protein (mg/dL)	49	11	<6.1

Since its presentation and temporal evolution highly suggested toxidermia, the hypothesis of ED was put forward. Nitrofurantoin was immediately stopped, and the patient was treated with intravenous fluids, topical corticosteroid (beclometasone twice daily), oral antihistamine (hydroxyzine 25mg/day), and topical care (antiseptic baths and emollients). A favorable clinical evolution was verified within a week, with the resolution of all lesions.

## Discussion

The overuse of antibiotics is a universal phenomenon in daily medical practice. Most of the adverse dermatological reactions are skin rashes, pruritus, Steven-Johnson syndrome, and exfoliative dermatitis [5].

ED is a severe generalized inflammation of the skin, characteristically demonstrating diffuse redness, desquamation, erosion, and scab formation on epidermal folds and mucosa. Its classical finding is bright red patches that coalesce to cover the skin surface, and patients may complain of tight skin due to progressive edema and lichenification. Pruritus occurs in nearly all patients, and it could be associated with systemic symptoms like fever, shivering, and nausea [2,3,6]. Lymphadenopathy, splenomegaly, and hepatomegaly may be present in 50% of patients [2,7].

The pathogenesis of ED is unknown. Triggers can be grouped into several general categories, including preexisting inflammatory dermatosis, adverse drug reactions (Table 2), and malignancies (lymphoma, leukemia, and solid tumors). Few cases are idiopathic [2,6,8].

**Table 2:** Drugs most frequently reported as the cause of exfoliative dermatitis ACE - angiotensin-converting enzyme Sources: [2,5,9]

Drug most frequently reported	
ACE inhibitors	Captopril, enalapril, lisinopril
Antibiotics	Ciprofloxacin, penicillins, streptomycin, sulfonamids, trimethoprim sulfamethoxazole, vancomycin
Antiepileptic	Carbamazepine, phenobarbital, phenytoin
Antitubercular	Isoniazid, rifampicin
Proton pump inhibitors	Omeprazole, esomeprazole, pantoprazole
Retinoids	Acitretin, isotretinoin
Other drugs	Allopurinol, chlorpromazine, dapsone, diltiazem, hydroxychloroquine, lithium, sertraline, sulfasalazine, terbinafine

ED is a clinical diagnosis based on dermatological findings. Laboratory studies, although nonspecific, and histopathology can help in identifying an underlying cause [1,2].

Being a potentially life-threatening state of "skin failure", initial management includes monitoring and ensuring metabolic and hemodynamic stability since patients are at risk of hypothermia, fluid and electrolyte imbalance, and secondary infections [2,3,6].

Treatment evolves symptom management and appropriate wound care with a lukewarm bath, topical corticosteroids, and bland emollients. Oral antihistamines may be helpful. Routine use of systemic antibiotics is not recommended [8]. Once identified, proper treatment of the underlying cause is mandatory. Drug-associated ED resolves quickly after discontinuation of the offending [6-9].

## Conclusions

ED is a rare clinical syndrome that may have a guarded prognosis. While its diagnosis can be established by physical examination, based on dermatological findings, it is essential to determine its etiology in order to start prompt and appropriate treatment.

Adverse drug reactions represent its second leading cause and, although several drugs have been implicated, to the best of our knowledge, nitrofurantoin-induced ED has not been previously reported. It is important to know and recognize this condition given that, after the withdrawal of the offending agent, long-term prognosis for patients with drug-induced disease is good.
